# Doctors are to blame for perceived medical adverse events. A cross sectional population study. The Tromsø study

**DOI:** 10.1186/1472-6963-13-46

**Published:** 2013-02-05

**Authors:** Ragnar Hotvedt, Olav Helge Førde

**Affiliations:** 1 Department of Community Medicine, Faculty of Health Sciences, University of Tromsø, Tromsø 9037, Norway

**Keywords:** Medical adverse events, Medical errors, Patient safety, Medical negligence, Occurrence

## Abstract

**Background:**

Most current knowledge of the incidence of medical adverse events (AEs) comes from studies carried out in hospital settings. Little is known about AEs occurring outside hospitals, in spite the fact that most of contacts between patients and health care take place in primary care. Small sample population studies report that 4–49% of the general public have experienced AEs related to their own or family members´ care.

The purpose with the present study was to investigate the occurrence of experienced medical adverse events in a large general population.

**Methods:**

We invited 19763 inhabitants of a municipality in northern Norway, age 30 years and older, to fill in a questionnaire. Main outcome measures were life time prevalence of AEs experienced by respondents or their first degree relatives, perceived responsibility for and predictors of such events, as well as formal complaints as a reaction to the events.

**Results:**

The response rate was 66%. Nine and 10% of the respondents reported self-experienced adverse events, and 15 and 19% (men and women, respectively) that their relatives had experienced AEs. Logistic regression models showed that the strongest predictors of reporting self-experienced adverse events were: Having been persuaded to accept an unwanted examination or treatment, difficulties in getting a referral from primary to specialist health care, and inadequate communication with the doctor. Of the respondents who had experienced adverse events personally, 62% placed the responsibility for the event on the general practitioner, 39% on the hospital doctor, and 19% on failing routines or cooperation. Only 7% of men and 14% of women who reported self-experienced events handed in a formal complaint.

**Conclusions:**

The public predominantly place the responsibility for medical adverse events on doctors, in particular general practitioners, and to a lesser degree on the system. This should be emphasised by doctors and managers who communicate with patients who have experienced AEs, and in patient safety work. Only a small fraction of adverse events results in a formal written complaint. Therefore, such complaints are of limited value as a basis for patient safety work.

## Background

Since the Institute of Medicine report *To Err Is Human*[1], medical adverse events (AEs) have been increasingly focused. In the efforts to improve patient safety by reducing the occurrence of AEs, a main strategy has been to consider the health care as a system, with inherent system errors that can be corrected, rather than blaming the individual fallible health worker.

Recording AEs are fundamental in monitoring effects of patient safety measures. Despite the fact that most contact between patients and the health system takes place in community-based primary care, most current knowledge of the incidence of AEs is based on studies from hospital settings. It has been estimated that AEs occur in 3 – 17% of hospital admissions [2-8]. Comparatively little is known about AEs outside hospitals. Population studies based on relatively small samples, and often with a low response rate, report that 4 – 49% of the general public have experienced AEs related to their own or family members´ care [9-13]. In a recent consumer survey from the European Commission, on average 26% of the respondents claim that they themselves or a member of their family had experienced an AE when receiving health care, with a variation among the European Union Member States from 12 to 49% [13].

In the present study, we asked inhabitants in a Norwegian municipality, age 30 years and older, whether they themselves or their near relatives had ever experienced a medical adverse event, and in case, where the responsibility for the event should be placed. We also identified personal and other characteristics associated with reporting such events.

## Methods

The Tromsø Study was initiated in 1974, and is a population-based study of inhabitants in the municipality of Tromsø, Norway. Tromsø has 65.286 inhabitants (January 2008), of which 38.440 are aged 30 – 87 years. The municipality is roughly representative for Norway regarding parameters like unemployment, personal income, proportions living in urban areas and number of General Practitioners per 10.000 residents [14]. The education level is higher, and Tromsø hosts the University Hospital of Northern Norway, which also serves as a local hospital for the municipality.

The Tromsø Study has a design which includes repeated population health surveys to which total birth cohorts and random samples are invited. The sixth survey, from which data in the present study is retrieved, was conducted in 2007 – 2008. The study was approved by the Data Inspectorate of Norway and the Regional Committee of Medical and Health Research Ethics, North Norway. The study complies with the Declaration of Helsinki, and each subject gave general written informed consent prior to participation in the Tromsø Study. Participation in the study was voluntary, and the participants had the right to refuse specific examinations whenever they chose.

Altogether 19763 persons, age 30 years and older, were invited to fill in a questionnaire (Additional file 1). In the questionnaire we included the following questions: “Have you ever experienced that diseases affecting yourself have been insufficiently examined or treated, resulting in serious consequences for you?” and “Have you ever experienced that diseases affecting your near relatives (child, parents, spouse) have been insufficiently examined or treated, resulting in serious consequences?” In case the respondents answered “yes” on one or both of these questions, we asked them to indicate where the responsibility for the event should be placed: general practitioner (GP), emergency GP, private specialist, hospital doctor, other health personnel, alternative medical practitioner or inadequate routines. The respondents were allowed to indicate perceived responsibility on more than one place. We also asked whether the respondents had considered complaining about the event, or whether they in fact had complained, orally or in writing.

The questionnaire further included questions concerning self-evaluated general health (1 = Very bad, 2 = Bad, 3 = Neither good or bad, 4 = Good, 5 = Excellent) (Additional file 2), marital status (1 = Single, 2 = Married, 3 = Widow/Widower, 4 = Divorced, 5 = Separated, 6 = Registered partnership, 7 = Separated partnership, 8 = Divorced partnership), level of education (1 = Primary/part secondary, 2 = Secondary, 3 = A-level, 4 = College/university less than 4 years, College/university 4 years or more), use of health services last years, whether the respondent had a hard time to understand what the doctor(s) said (Eleven categories, 0 = Very difficult to understand, 10 = Not difficult at all), and whether it had been a problem to be referred from a GP to specialist health care (1 = Very difficult, 2 = Some difficulties, 3 = Easy, 4 = Very easy). We also asked whether the respondents had ever felt persuaded to accept an examination or treatment which they did not want (yes/no), and in case if this had led to unfortunate consequences for their health (yes/no).

### Statistical analysis

Multiple logistic regression models were used to explore the associations between a set of background variables and adverse events and complaining as binary dependent variables. The following predictor variables were included in the models: Sex, age groups (ten years and twenty years), self-evaluated general health condition, level of education, marital status (categories 2 and 6 coded as Married, categories 1, 3 – 5, 7 and 8 as Single), understanding the GP (categories 0 – 4 coded as Difficult, categories 5 – 7 as Intermediate and categories 8 – 10 as Easy), and referral to specialist health care (categories 1 and 2 coded as Difficult, category 3 as Easy and category 4 as Very easy). Odds ratios and 95% confidence intervals were estimated.

To compare proportions among ordinal categories of data (age categories), we used Chi square test for trend.

All statistical analyses were performed with SPSS software. Two-sided p values were used, and p = < 0.05 was considered to indicate statistical significance.

## Results

Out of the invited inhabitants of Tromsø municipality, 12984 persons filled in the questionnaire, giving a response rate of 66%. The mean age was 57.5 years (range 30 – 87), and 53% were women. Eighty-three% of the respondents had consulted a GP during the last year, 35% had been examined or treated by the specialist health care, 12% had been admitted to a hospital, and 24% had undergone surgery during the last three years.

As shown in Table 1, 9% of men and 10% of women reported to have experienced adverse events themselves, and 15% of the men and 19% of the women reported that their near relatives had experienced adverse events.

**Table 1:** Reported adverse events by sex and age

**Age (years)**	**Men**			**Women**		
	**Number of persons N**	**Adverse events affecting myself n (%)**	**Adverse events affecting relatives n (%)**	**Number of persons n**	**Adverse events affecting myself n (%)**	**Adverse events affecting relatives n (%)**
30-39	195	11 (5.6)	41 (21.0)	272	28 (10.3)	58 (21.3)
40-49	1554	128 (8.2)	269 (17.3)	1794	164 (9.1)	410 (22.9)
50-59	1071	101 (9.4)	176 (16.4)	1193	105 (8.8)	247 (20.7)
60-69	1847	183 (9.9)	268 (14.5)	1906	190 (10.0)	317 (16.6)
70-79	762	93 (12.2)	68 (8.9)	818	92 (11.2)	110 (13.4)
80-87	160	12 (7.5)	15 (9.4)	253	37 (14.6)	22 (8.7)
Total	5589	528 (9.4)	837 (15.0)	6236	616 (9.9)	1164 (18.7)

Life time prevalence, total population 11825 persons.

Multiple logistic regression models showed that respondents who had felt persuaded to accept an examination or a treatment they did not want (4% of the respondents), or who had found it difficult to get a referral from primary to specialist health care (9% of the respondents), reported a markedly increased occurrence of self-experienced AEs and AEs affecting their relatives (Table 2). Respondents who found it difficult to understand what the GP said, or who had a low self-evaluated health, also more often reported both self-experienced AEs and AEs affecting their relatives.

**Table 2:** Multiple logistic regression model of factors associated with self-experienced adverse events and adverse events affecting relatives (n = 11116)

	**Dependent variable**			
	**Self-experienced adverse event**		**Adverse events affecting relatives**	
**Factors**	**Adjusted odds ratio**	**95% CI**	**Adjusted odds ratio**	**95% CI**
Age groups (1 = 30–39, 8 = 80–87)	1.11	1.05 – 1.17	.88	.84 – .92
Sex (0 = Female, 1 = Male)	1.06	.93 – 1.21	.80	.72 – .88
Marital status (1 = Married, 2 = Single)	1.23	1.08 – 1.41	1.05	.94 – 1.16
Education (1 = Primary/part secondary, 5 = College/university 4 years or more)	1.10	1.05 – 1.16	1.15	1.11 – 1.19
Self-evaluated health (1 = Very bad, 5 = Excellent)	.56	.51 – .61	.85	.80 – .91
Understanding GP (1 = Difficult, 3 = Easy)	.76	.65 – .89	.84	.73 – .96
Referral to specialist				
Difficult	2.81	2.24 – 3.53	1.92	1.59 – 2.34
Easy	1.15	.94 – 1.42	1.29	1.09 – 1.51
Very easy (reference)	1.00		1.00	
Persuaded treatment (0 = No, 1 = Yes)	3.63	2.86 – 4.59	1.47	1.17 – 1.85

*GP*, General practitioner.

Out of the 1124 respondents who indicated that AE had affected them, 62% were of the opinion that the GP was responsible for the AE, 39% that the hospital doctor was responsible, and 19% related the AEs to failing routines or cooperation (Table 3). Out of the respondents with self-experienced AEs, 14% placed the responsibility on two or more sources. Among the 1969 respondents indicating that AE had occurred to relatives, 58% placed the responsibility on the GP, 38% on the hospital doctor and 23% on failing routines or cooperation, 14% of these respondents placed the responsibility on two or more places. For respondents with self-experienced AEs who placed the responsibility on the GP, 9% also placed responsibility on failing routines. Respondents with self-experienced AEs who placed the responsibility on the hospital doctor, also placed responsibility on failing routines in 8% of the cases.

**Table 3:** Perceived responsibility for adverse events by age

	**Affecting myself**				**Affecting relatives**			
		**Responsible**				**Responsible**		
**Age (years)**	Adverse events n *	**GP n (%)**	**Hospital doctor n (%)**	**Failing routines n (%)**	Adverse events n *	**GP n (%)**	**Hospital doctor n (%)**	**Failing routines n (%)**
30-39	39	28 (71.8)	12 (30.8)	7 (17.9)	99	58 (58.6)	37 (37.4)	27 (27.3)
40-49	288	181 (62.8)	98 (34.0)	47 (16.3)	670	407 (60.7)	238 (35.5)	144 (21.5)
50-59	201	137 (68.2)	78 (38.8)	44 (21.9)	414	238 (57.5)	166 (40.1)	101 (24.4)
60-69	368	212 (57.6)	160 (43.5)	72 (19.6)	577	324 (56.2)	237 (41.1)	140 (24.3)
70-79	180	107 (59.4)	70 (38.9)	34 (18.9)	174	98 (56.3)	63 (36.2)	38 (21.8)
80-87	48	30 (62.5)	20 (41.7)	5 (10.4)	35	23 (65.7)	11 (31.4)	8 (22.9)
Total	1124	695 (61.8)	438 (39.0)	209 (18.6)	1969	1148 (58.3)	752 (38.2)	458 (23.3)
Chi square for trend, p value		0.094	0.045	0.996		0.262	0.376	0.842

* = Number of persons reporting adverse events and perceived responsibility.

For respondents who reported self-experienced AEs and considered the hospital doctor to be responsible, we found a significant trend of increasing doctor-responsibility with increasing age (Table 3). For the other responsibility categories we found no statistically significant trends among the age groups.

Respondents who reported self-experienced AEs were asked if they had considered complaining as a reaction to the events. Among men, 42% reported that they had considered complaining, 27% that they had complained orally, and 7% that they had complained formally in writing (Table 4). Among women, the proportions were 40, 28 and 14%, respectively.

**Table 4:** Complaining as a reaction to self-experienced adverse events

	**Number of persons n**		**Complaining considered n (%)**		**Complained orally n (%)**		**Complained in writing n (%)**	
**Age (years)**	**Men**	**Women**	**Men**	**Women**	**Men**	**Women**	**Men**	**Women**
30-49	137	191	61 (44.5)	81 (42.4)	31 (22.6)	46 (24.1)	7 (5.1)	24 (12.6)
50-69	275	288	108 (39.3)	112 (38.9)	88 (32.0)	84 (29.2)	25 (9.1)	43 (14.9)
70-87	100	125	45 (45.0)	51 (40.8)	21 (21.0)	39 (31.2)	6 (6.0)	17 (13.6)
Total	512	604	214 (41.8)	244 (40.4)	140 (27.3)	169 (28.0)	38 (7.4)	84 (13.9)

Data from 1116 persons by sex and age.

In multiple logistic regression analyses we found no significant associations between complaining and the included predictors (Table 5), with two exceptions: Respondents who reported it difficult to be referred to specialist health care were more likely to consider complaining, and women were more likely to complain in writing than men.

**Table 5:** Multiple logistic regression model of factors associated with complaining

	**Dependent variable**			
	**Complaining considered**		**Complained in writing**	
**Factors**	**Adjusted odds ratio**	**95% CI**	**Adjusted odds ratio**	**95% CI**
Age groups (1 = 30–49, 3 = 70–87)	.97	.80 – 1.18	1.20	.88 – 1.62
Sex (0 = Female, 1 = Male)	1.09	.84 – 1.40	.51	.33 – .78
Marital status (1 = Married, 2 = Single)	.82	.63 – 1.06	1.46	.98 – 2.19
Education (1 = Primary/part secondary, 5 = College/university 4 years or more)	1.06	.97 – 1.15	1.05	.91 – 1.20
Self-evaluated health (1 = Very bad, 5 = Excellent)	1.01	.86 – 1.17	.96	.75 – 1.22
Understanding GP (1 = Difficult, 3 = Easy)	.79	.60 – 1.05	1.20	.74 – 1.96
Referral to specialist				
Difficult	1.58	1.03 – 2.43	1.00	.52 – 1.95
Easy	1.47	.98 – 2.22	1.03	.55 – 1.92
Very easy (reference)	1.00		1.00	
Persuaded treatment (0 = No, 1 = Yes)	1.17	.80 – 1.71	1.25	.71 – 2.21

Data from 1042 persons reporting self-experienced adverse events.

Out of the total number of respondents included in the present study, 253 respondents (2%) reported that they ever had complained in writing to the health services. But only 48% of these respondents stated that they had experienced an AE themselves, and 29% that an AE had occurred to their relatives. The rest of the written complaints are related to other circumstances than experienced AEs.

## Discussion

Approximately 10% of our almost twelve thousand respondents stated that they personally ever had experienced an AE, and 15–19% that an AE had affected their near relatives. Taken into consideration that this is a life time cumulative incidence, it is a considerably lower incidence than would be expected from data in small samples population studies, partly recorded during limited time periods [10-13]. The lower incidence of AEs found in our study can be explained by recall bias and the fact that most AEs are minor and cause only temporary symptoms [2,4-6,9]. Major adverse events may be remembered for life, but minor events may be remembered only if occurred recently. On the other hand, our study population obviously includes some first degree relatives. Therefore, some respondents may report the same adverse event occurring in the same relative. This would, to a minor degree, inflate the estimates of prevalence of adverse events reported for relatives.

The strength of our study is the large representative sample obtained from a general population, and a high response rate. Further, this study, in contrast to many other studies of medical adverse events or medical errors, also includes the public’s experience from contacts with the primary health care, as more than four out of five of our respondents reported to have consulted a GP last year and consequently the majority of the reported AEs were related to the GP.

The present study, as well as other studies exploring incidence and causes of adverse events, has an important methodological problem: The identification of a medical error, a mishap or an adverse event is a subjective process. This is true for lay people as well as health professionals. Attempts to reduce the problem have been done by introducing definitions [1,15], examples [9,12] and improved taxonomies [16], but the problem still persists. Some authors have focused on challenges especially related to primary care [17-19]. In our study, we have not defined the concept “adverse event”. We have only asked whether the respondents have experienced that insufficient examination or treatment has had “serious consequences”. Their answers therefore include all kinds of unexpected outcomes. A deeper exploration into these challenges needs further studies.

We found that respondents who had felt persuaded to accept an examination or a treatment they did not want, and respondents who had found it difficult to be referred from a GP to specialist health care, had a markedly increased likelihood to report AEs, affecting themselves as well as their relatives. This probably reflects aspects of the patient-doctor relation: A more consumer-like attitude from patients, with increased expectations of easy access and a perfect outcome, combined with strengthened patient rights, and increasing demands for service will inevitably increase the occurrence of perceived AEs.

As an indicator of communication problems between patient and doctor, we asked the respondents if they understood what the doctor said during the last consultation. We found that such communication difficulties strongly increased the probability for reporting AEs. This is in accordance with other studies [20,21], and indicates that doctors should aim for better communication skills [22].

Respondents reporting to have experienced an AE, occurring to themselves or their relatives, stated that their GP was responsible for the AE in about 60% of the cases, hospital doctors in about 40%, and the system (failing routines or communication) in 20%. This distribution of perceived responsibility probably in part reflects the frequent contact between the respondents and the primary health care, but also indicate that patients interact and relate more to individual health professionals, in particular doctors, than to systems. Our results are in line with studies by Blendon et al. [12] and Northcott et al. [10], who found that members of the public, as well as doctors, were more likely to place the responsibility for medical errors on individual health professionals than on the institution involved. Doctors, management and superior authority who are confronted by patients or public to discuss AEs should consider the distribution of perceived responsibility found in our study, even if the distribution may be felt unfair.

When strategies for quality improvement in health care systems are elaborated, the prevailing view is often that adverse events primarily are caused by failures of institutional systems. This perception of reality is not supported by the present population. Positive effects of system based interventions to improve patient safety have only modest supporting evidence [23]. In their daily work doctors may tend not to adhere to protocols, and to be reluctant to use standardised routines [24]. In patient safety work, where avoiding “blaming and shaming” and search for scapegoats is part of the recommended strategy, the personal responsibility of doctors and of other health professionals still should be emphasised.

Although about 40% of the respondents having experienced AEs personally considered complaining about the events, only about 10% in fact did so in writing. It has also been shown by others that AEs rarely lead to complaining, and very few medical errors result in medical-malpractice claims [25]. Norway and other countries have several complex and often uncoordinated systems for recording and handling of patient complaints. In Norway, a country with about 5 million inhabitants, a so called no-blame system, “The Norwegian System of Compensation to Patients” [26], was established in 1988. This system, intended to be a low threshold system, receives compensation claims from patients treated in both public and private health services. In 2010, the system received 4352 claims from patients who had experienced an AE. Seventy eight % of the patients were older than 30 years of age, and 32% of them achieved economic compensation.

Further, it should be noticed that only approximately 50% of the respondents who reported to have complained in writing in the present study, reported to have experienced AEs personally, and about 30% of them that AEs had occurred to their relatives. This means that many complaints are caused by other factors than experienced AEs, as communication problems or substandard professional behaviour. Altogether, this implies that patient complaints are of limited value as a basis for efforts to reduce the incidence of AEs.

## Conclusion

In this population study, about 10% of the respondents stated that they ever had experienced a medical adverse event themselves, and 15–19% that an AE had occurred to their relatives. The respondents mainly place the responsibility for AEs on doctors, in particular on general practitioners, and to a lesser degree on system failures. This should be emphasised by doctors and managers who communicate with patients who have experienced AEs, and in patient safety work. Only a small fraction of perceived AEs results in a written complaint. Therefore, written complaints are of limited value as a basis for patient safety work.

## Competing interests

The authors declare that they have no competing interests.

## Authors’ contributions

Both authors initiated and designed the study, and performed the statistical analyses and interpretation of data. RH has been the primary author of the manuscript, while OHF has revised it and contributed to the writing. Both authors read and approved the final manuscript.

## Pre-publication history

The pre-publication history for this paper can be accessed here:

http://www.biomedcentral.com/1472-6963/13/46/prepub

## Supplementary Material

Additional file 1 **Questionnaire.** Mailed together with the invitation to participate in the Tromsø Study (Tromsø 6). Includes questions about general health status, use of health services and medication, socioeconomic situation etc. Click here for file

Additional file 2 **Extended questionnaire.** Delivered to persons attending the study. Click here for file
