# Supplementary material for: Doctors are to blame for perceived medical adverse events. A cross sectional population study. The Tromsø study
Source: BMC Health Serv Res. 2013 Feb 5;13:46. doi: 10.1186/1472-6963-13-46 (PMC3623705; doi:10.1186/1472-6963-13-46)
Supplement: Additional file 1 — Questionnaire. Mailed together with the invitation to participate in the Tromsø Study (Tromsø 6). Includes questions about general health status, use of health services and medication, socioeconomic situation etc. [file 1472-6963-13-46-S1.pdf]

The Tromsø Study

The form will be read electronically. Please use a blue or black pen

You can not use comas, use upper-case letters.

2007 - 2008 Confidential

HEALTH AND DISEASES

1 **How do you in general consider your own health to be?**

- ☐ Very good
- ☐ Good
- ☐ Neither good nor bad
- ☐ Bad
- ☐ Very bad

2 **How is your health compared to others in your age?**

- ☐ Much better
- ☐ A little better
- ☐ About the same
- ☐ A little worse
- ☐ Much worse

3 **Do you have, or have you had?**

	Yes	No	Age first time
A heart attack	☐	☐	
Angina pectoris *(heart cramp)*	☐	☐	
Cerebral stroke/brain hemorrhage	☐	☐	
Atrial fibrillation	☐	☐	
High blood pressure	☐	☐	
Osteoporosis	☐	☐	
Asthma	☐	☐	
Chronic bronchitis/Emphysema/COPD	☐	☐	
Diabetes	☐	☐	
Psychological problems *(for which you have sought help)*	☐	☐	
Hypothyroidism	☐	☐	
Kidney disease, *not including urinary tract infection (UTI)*	☐	☐	
Migraine	☐	☐	

4 **Do you have persistent or constantly recurring pain that has lasted for 3 months or more?**

☐ Yes ☐ No

5 **How often have you suffered from sleeplessness during the last 12 months?**

- ☐ Never, or just a few times
- ☐ 1-3 times a month
- ☐ Approximately once a week
- ☐ More that once a week

6 **Below you find a list of various problems. Have you experienced any of this during the last week (including today)?** (Tick once for each complaint)

	No complaint	Little complaint	Pretty much	Very much
Sudden fear without reason	☐	☐	☐	☐
Felt afraid or anxious	☐	☐	☐	☐
Faintness or dizziness	☐	☐	☐	☐
Felt tense or upset	☐	☐	☐	☐
Tend to blame yourself	☐	☐	☐	☐
Sleeping problems	☐	☐	☐	☐
Depressed, sad	☐	☐	☐	☐
Feeling of being useless, worthless	☐	☐	☐	☐
Feeling that everything is a struggle	☐	☐	☐	☐
Feeling of hopelessness with regard to the future	☐	☐	☐	☐

USE OF HEALTH SERVICES

7 **Have you during the last 12 months visited:**
If YES; how many times?

	Yes	No	No. of times
General practitioner (GP)	☐	☐	
Psychiatrist/psychologist	☐	☐	
Medical specialist outside hospital *(other than general practitioner/psychiatrist)*	☐	☐	
Physiotherapist	☐	☐	
Chiropractor	☐	☐	
Alternative practitioner *(homeopath, acupuncturist, foot zone therapist, herbal medicine practitioner, laying on hands practitioner, healer, clairvoyant, etc.)*	☐	☐	
Dentist/dental service	☐	☐	

8 **Have you during the last 12 months been to a hospital?**

	Yes	No	No. of times
Admitted to a hospital	☐	☐	
Had consultation in a hospital without admission;			
At psychiatric out-patient clinic	☐	☐	
At another out-patient clinic	☐	☐	

9 **Have you undergone any surgery during the last 3 years?**

☐ Yes ☐ No

USE OF MEDICINES

10 **Do you currently use, or have you used some of the following medicines?** (Tick once for each line)

	Never used	Now	Earlier	Age first time
Blood pressure lowering drugs	☐	☐	☐	
Cholesterol lowering drugs	☐	☐	☐	
Drugs for heart disease	☐	☐	☐	
Diuretics	☐	☐	☐	
Drugs for osteoporosis	☐	☐	☐	
Insulin	☐	☐	☐	
Tablets for diabetes	☐	☐	☐	
The drugs for hypothyroidism Thyroxine/levaxin	☐	☐	☐	

11 **How often have you during the last 4 weeks used the following medicines?** (Tick once for each line)

	Not used in the last 4 weeks	Less than every week	Every week, but not daily	Daily
Painkillers on prescription	☐	☐	☐	☐
Painkillers non-prescription	☐	☐	☐	☐
Sleeping pills	☐	☐	☐	☐
Tranquillizers	☐	☐	☐	☐
Antidepressants	☐	☐	☐	☐

12 **State the name of all medicines -both those on prescription and non-prescription drugs- you have used regularly during the last 4 weeks.**
Do not include vitamins, minerals, herbs, natural remedies, other nutritional supplements, etc.

If there is not enough space for all medicines, continue on a separate sheet.

When attending you will be asked whether you have used antibiotics or painkillers the last 24 hours. If you have, you will be asked to provide the name of the drug, strength, dose and time of use.

FAMILY AND FRIENDS

13 **Who do you live with?** (Tick for each question and give the number)

	Yes	No	Number
Spouse/partner	☐	☐	
Other people older than 18 years	☐	☐	
People younger than 18 years	☐	☐	

14 **Tick for the relatives who have or have had**

	Parents	Children	Siblings
A heart attack	☐	☐	☐
A heart attack before age of 60	☐	☐	☐
Angina pectoris *(heart cramp)*	☐	☐	☐
Cerebral stroke/brain haemorrhage	☐	☐	☐
Osteoporosis	☐	☐	☐
Gastric/duodenal ulcers	☐	☐	☐
Asthma	☐	☐	☐
Diabetes	☐	☐	☐
Dementia	☐	☐	☐
Psychological problems	☐	☐	☐
Substance abuse	☐	☐	☐

15 **Do you have enough friends who can give you help when you need it?**

☐ Yes ☐ No

16 **Do you have enough friends whom you can talk confidentially with?**

☐ Yes ☐ No

17 **How often do you normally take part in organised gatherings, e.g. sport clubs, political meetings, religious or other associations?**

- ☐ Never, or just a few times a year
- ☐ 1-2 times a month
- ☐ Approximately once a week
- ☐ More than once a week

WORK, SOCIAL SECURITY AND INCOME

18 **What is the highest level of education you have completed?** (Tick once)

- ☐ Primary/secondary school, modern secondary school
- ☐ Technical school, vocational school, 1-2 years senior high school
- ☐ High school diploma
- ☐ College/university less than 4 years
- ☐ College/university 4 years or more

19 **What is your main activity?** (Tick once)

- ☐ Full time work
- ☐ Part time work
- ☐ Unemployed
- ☐ Housekeeping
- ☐ Retired/benefit recipient
- ☐ Student/military service

20 **Do you receive any of the following benefits?**

- ☐ Old-age, early retirement or survivor pension
- ☐ Sickness benefit (on sick leave)
- ☐ Rehabilitation benefit
- ☐ Full disability pension
- ☐ Partial disability pension
- ☐ Unemployment benefits
- ☐ Transition benefit for single parents
- ☐ Social welfare benefits

21 **What was the household's total taxable income last year?** Include income from work, pensions, benefits and similar

- ☐ Less than 125 000 NOK
- ☐ 125 000-200 000 NOK
- ☐ 201 000-300 000 NOK
- ☐ 301 000-400 000 NOK
- ☐ 401 000-550 000 NOK
- ☐ 551 000-700 000 NOK
- ☐ 701 000 -850 000 NOK
- ☐ More than 850 000 NOK

22 **Do you work outdoor at least 25% of the time, or in cold buildings (e.g. storehouse/industry buildings)?**

☐ Yes ☐ No

PHYSICAL ACTIVITY

23 **<u>If</u> you have paid or unpaid work, which statement describes your work best?**

- ☐ Mostly sedentary work
  (e.g. office work, mounting)
- ☐ Work that requires a lot of walking
  (e.g. shop assistant, light industrial work, teaching)
- ☐ Work that requires a lot of walking and lifting
  (e.g. postman, nursing, construction)
- ☐ Heavy manual labour

24 **Describe your exercise and physical exertion in leisure time. If your activity varies much, e.g. between summer and winter, then give an average. The question refers only to <u>the last year</u>.** (Tick the most appropriate box)

- ☐ Reading, watching TV, or other sedentary activity.
- ☐ Walking, cycling, or other forms of exercise at least 4 hours a week *(include walking or cycling to work, Sunday-walk/stroll, etc.)*
- ☐ Participation in recreational sports, heavy gardening, etc. *(note:duration of activity at least 4 hours a week)*
- ☐ Participation in hard training or sports competitions, regularly several times a week.

25 **How often do you exercise?** (With exercise we mean for example walking, skiing, swimming or training/sports)

- ☐ Never
- ☐ Less than once a week
- ☐ Once a week
- ☐ 2-3 times a week
- ☐ Approximately every day

26 **How hard do you exercise on average?**

- ☐ Easy- do not become short-winded or sweaty
- ☐ You become short-winded and sweaty
- ☐ Hard- you become exhausted

27 **For how long time do you exercise every time on average?**

- ☐ Less than 15 minutes
- ☐ 15-29 minutes
- ☐ 30-60 minutes
- ☐ More than 1 hour

ALCOHOL AND TOBACCO

28 **How often do you drink alcohol?**

- ☐ Never
- ☐ Monthly or less frequently
- ☐ 2-4 times a month
- ☐ 2-3 times a week
- ☐ 4 or more times a week

29 **How many units of alcohol** (a beer, a glass of wine or a drink) **do you usually drink when you drink alcohol?**

- ☐ 1-2
- ☐ 3-4
- ☐ 5-6
- ☐ 7-9
- ☐ 10 or more

30 **How often do you drink 6 units of alcohol or more in one occasion?**

- ☐ Never
- ☐ Less frequently than monthly
- ☐ Monthly
- ☐ Weekly
- ☐ Daily or almost daily

31 **Do you smoke sometimes, but not daily?**

☐ Yes ☐ No

32 **Do you/did you smoke daily?**

☐ Yes, now ☐ Yes, previously ☐ Never

33 **If you previously smoked daily, how long is it since you quit?**

Number of years ☐☐

34 **If you currently smoke, or have smoked previously: How many cigarettes do you or did you usually smoke per day?**

Number of cigarettes ☐☐

35 **How old were you when you began daily smoking?**

Age in years ☐☐

36 **How many years in all have you smoked daily?**

Number of years ☐☐

37 **Do you use or have you used snuff or chewing tobacco?**

- ☐ No, never
- ☐ Yes, previously
- ☐ Yes, sometimes
- ☐ Yes, daily

DIET

38 **Do you usually eat breakfast every day?**

☐ Yes ☐ No

39 **How many units of fruit or vegetables do you eat on average per day?** (units means for example a fruit, a cup of juice, potatoes, vegetables)

Number of units ☐

40 **How many times a week do you eat warm dinner?**

Number ☐

41 **How often do you usually eat these foods?**
(Tick once for each line)

	0-1 times/ mth	2-3 times/ mth	1-3 times/ week	4-6 times/ week	1-2 times/ day
Potatoes	☐	☐	☐	☐	☐
Pasta/rice	☐	☐	☐	☐	☐
Meat *(not processed)*	☐	☐	☐	☐	☐
Processed meat *(sausages, hamburger, etc.)*	☐	☐	☐	☐	☐
Fruits, vegetables, berries	☐	☐	☐	☐	☐
Lean fish	☐	☐	☐	☐	☐
Fatty fish *(e.g. salmon, trout, mackerel, herring, halibut, redfish)*	☐	☐	☐	☐	☐

42 **How much do you usually drink the following?**
(Tick once for each line)

	Rarely/ never	1-6 glasses /week	1 glass /day	2-3 glasses /day	4 or more glasses /day
Milk, curdled milk, yoghurt	☐	☐	☐	☐	☐
Juice	☐	☐	☐	☐	☐
Soft drinks with sugar	☐	☐	☐	☐	☐

43 **How many cups of coffee and tea do you drink daily?** (Put 0 for the types you do not drink daily)

	Number of cups
Filtered coffee	☐
Boiled coffee (coarsely ground coffee for brewing)	☐
Other types of coffee	☐
Tea	☐

44 **How often do you usually eat cod liver and roe?**
(i.e. "mølje")

☐ Rarely/never ☐ 1-3 times/year ☐ 4-6 times/year
☐ 7-12 times/year ☐ More than 12 times/year

45 **Do you use the following nutritional supplements?**

	Daily	Sometimes	No
Cod liver oil or fish oil capsules	☐	☐	☐
Omega 3 capsules *(fish oil, seal oil)*	☐	☐	☐
Calcium tablets	☐	☐	☐

QUESTIONS FOR WOMEN

46 **Are you pregnant at the moment?**

☐ Yes ☐ No ☐ Uncertain

47 **How many children have you given birth to?**

Number ☐

48 **If you have given birth, fill in for each child: birth year, birth weight and months of breastfeeding** (Fill in the best you can)

Child	Birth year	Birth weight in grams	Months of breastfeeding
1	☐	☐	☐
2	☐	☐	☐
3	☐	☐	☐
4	☐	☐	☐
5	☐	☐	☐
6	☐	☐	☐

49 **Have you during pregnancy had high blood pressure?**

☐ Yes ☐ No

50 **If yes, during which pregnancy?**

☐ The first ☐ Second or later

51 **Have you during pregnancy had proteinuria?**

☐ Yes ☐ No

52 **If yes, during which pregnancy?**

☐ The first ☐ Second or later

53 **Were any of your children delivered prematurely (a month or more before the due date) because of preeclampsia?**

☐ Yes ☐ No

54 **If yes, which child?**

1st child	2nd child	3rd child	4th child	5th child	6th child
☐	☐	☐	☐	☐	☐

55 **How old were you when you started menstruating?**

Age ☐

56 **Do you currently use any prescribed drug influencing the menstruation?**

Oral contraceptives, hormonal intrautrine or similar	☐ Yes	☐ No
Hormone treatment for menopausal problems	☐ Yes	☐ No

When attending you will get supplementary questions about menstruation and any use of hormones. Write down on a sheet of paper the names of all the hormones you have used and bring it with you. You will also be asked whether your menstruation have ceased and possibly when and why.
