# Supplementary material for: Doctors are to blame for perceived medical adverse events. A cross sectional population study. The Tromsø study
Source: BMC Health Serv Res. 2013 Feb 5;13:46. doi: 10.1186/1472-6963-13-46 (PMC3623705; doi:10.1186/1472-6963-13-46)

Tromsø 6

- part of The Tromsø Study

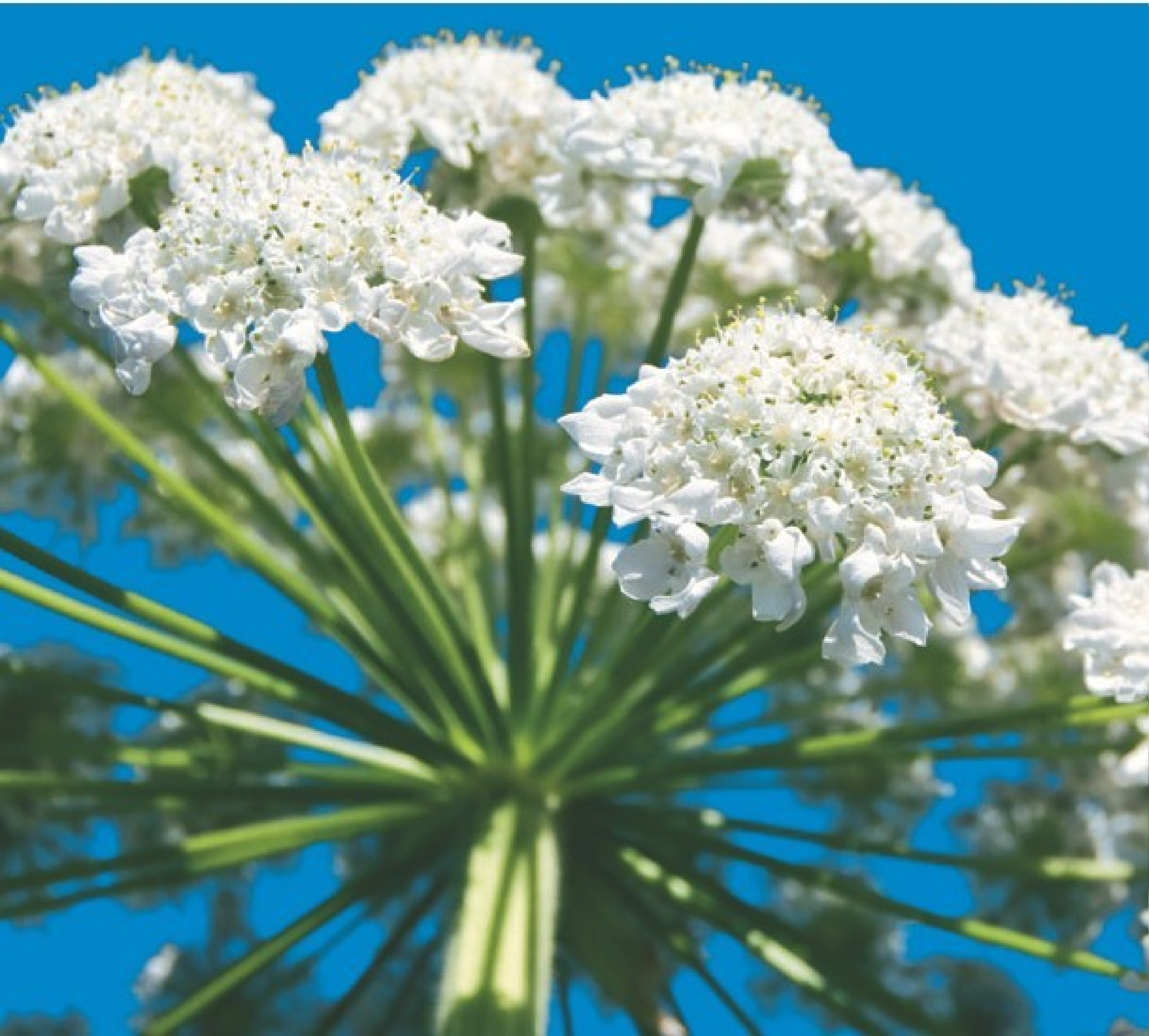

FILL OUT THE FORM IN THIS WAY:

The form would be read by machine, it is therefore important that you tick appropriately:

Correct

Wrong

Wrong

If you tick the wrong box, correct by filling the box like this

Write the numbers clearly 1 2 3 4 5 6 7 8 9 0

7,4 Correct

7,4 Wrong

Use only black or blue pen, do not use pencil or felt tip pen

1. DESCRIPTION OF YOUR HEALTH STATUS

By placing a tick in one box in each group below, please indicate which statements best describe your own health state today:

1.01 **Mobility**

- ☐ I have no problems in walking about
- ☐ I have some problems in walking about
- ☐ I am confined to bed

1.02 **Self-care**

- ☐ I have no problems with self-care
- ☐ I have some problems washing or dressing myself
- ☐ I am unable to wash or dress myself

1.03 **Usual activities** (e.g. work, study, housework, family or leisure activities)

- ☐ I have no problems with performing my usual activities
- ☐ I have some problems with performing my usual activities
- ☐ I am unable to perform my usual activities

1.04 **Pain and discomfort**

- ☐ I have no pain or discomfort
- ☐ I have moderate pain or discomfort
- ☐ I have extreme pain or discomfort

1.05 **Anxiety and depression**

- ☐ I am not anxious or depressed
- ☐ I am moderately anxious or depressed
- ☐ I am extremely anxious or depressed

1.6 To allow you to show us how good or bad your state of health is we have made a scale (almost like a thermometer) where the best state of health you can imagine is marked 100 and the worst 0. We ask you to show your state of health by drawing a line from the box below to the point on the scale that best fits your state of health.

Your own health state today

Best imaginable health state

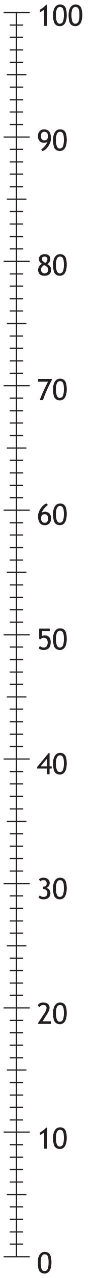

Worst imaginable health state

2. CHILDHOOD/YOUTH AND AFFILIATION

2.01 **Where did you live at the age of 1 year?**

- ☐ In Tromsø *(with present municipal borders)*
- ☐ In Troms, but not Tromsø
- ☐ In Finnmark
- ☐ In Nordland
- ☐ Another place in Norway
- ☐ Abroad

2.02 **How was your family's financial situation during your childhood?**

- ☐ Very good
- ☐ Good
- ☐ Difficult
- ☐ Very difficult

2.03 **What is the importance of religion in your life?**

- ☐ Very important
- ☐ Somewhat important
- ☐ Not important

2.04 **What do you consider yourself as?** (Tick for one or more alternatives)

- ☐ Norwegian
- ☐ Sami
- ☐ Kven/Finnish
- ☐ Another

2.05 **How many siblings and children do you have/have you had?**

Number of siblings .... ☐☐

Number of children .... ☐☐

2.06 **Is your mother alive?**

☐ Yes ☐ No

If NO: her age when she died .... ☐☐

Is your father alive?

☐ Yes ☐ No

If NO: his age when he died .... ☐☐

2.07 **What was/is the highest completed education for your parents and your spouse/partner?**
(Tick once for each column)

	Mother	Father	Spouse/ partner
7-10 years primary/secondary school, modern secondary school	☐	☐	☐
Technical school, vocational school, 1-2 years senior high school	☐	☐	☐
High school diploma	☐	☐	☐
College or university (less than 4 years)	☐	☐	☐
College or university (4 years or more)	☐	☐	☐

3. WELL BEING AND LIVING CONDITIONS

3.01 **Below are three statements about satisfaction with life as a whole. Then there are two statements about views on your own health. Show how you agree or disagree with each of the statements by ticking in the box for the number you think fits best for you.** (tick once for each statement)

	Completely disagree 1	2	3	4	5	6	7 Completely agree
In most ways my life is close to my ideal	☐	☐	☐	☐	☐	☐	☐
My life conditions are excellent	☐	☐	☐	☐	☐	☐	☐
I am satisfied with my life	☐	☐	☐	☐	☐	☐	☐
I have a positive view of my future health	☐	☐	☐	☐	☐	☐	☐
By living healthy, I can prevent serious diseases	☐	☐	☐	☐	☐	☐	☐

3.02 **Below are four statements concerning your current job conditions, or if you are not working now, the last job you had.** (Tick once for each statement)

	Completely disagree 1	2	3	4	5	6	7 Completely agree
My work is tiring, physically or mentally	☐	☐	☐	☐	☐	☐	☐
I have sufficient influence on when and how my work should be done	☐	☐	☐	☐	☐	☐	☐
I am being bullied or harassed at work	☐	☐	☐	☐	☐	☐	☐
I am being treated fairly at work	☐	☐	☐	☐	☐	☐	☐

3.03 **I consider my occupation to have the following social status in the society** (if you are not currently employed, think about your latest occupation)

- ☐ Very high status
- ☐ Fairly high status
- ☐ Medium status
- ☐ Fairly low status
- ☐ Very low status

3.04 **Have you over a long period experienced any of the following?** (Tick one or more for each line)

	No	Yes, as a child	Yes, as adult	Yes, last year
Been tormented, or threatened with violence	☐	☐	☐	☐
Been beaten, kicked at or victim of other types of violence	☐	☐	☐	☐
Someone in your close family have used alcohol or drugs in such a way that it has caused you worry	☐	☐	☐	☐

If you have experienced anything of the above, how much are you affected by that now?

☐ Not affected ☐ Affected to some extent ☐ Affected to a large extent

4. ILLNESS AND WORRIES

4.01 **Have you during the <u>last month</u> experienced any illness or injury?**

☐ Yes ☐ No

If YES: have you during the same period? (Tick once for each line)

	Yes	No
Been to a general practitioner	☐	☐
Been to a medical specialist	☐	☐
Been to emergency department	☐	☐
Been admitted to a hospital	☐	☐
Been to an alternative practitioner *(chiropractor, homeopath or similar)*	☐	☐

4.02 **Have you noticed sudden changes in your pulse or heart rythm in the <u>last year</u>?**

☐ Yes ☐ No

4.03 **Do you become breathless in the following situations?** (tick once for each question)

	Yes	No
When you walk rapidly on level ground or up a moderate slope	☐	☐
When you walk calmly on level ground	☐	☐
While you are washing or dressing	☐	☐
At rest	☐	☐

4.04 **Do you cough about daily for some periods of the year?**

☐ Yes ☐ No

If YES: Is the cough usually productive?

☐ Yes ☐ No

Have you had this kind of cough for as long as 3 months in each of the last two years?

☐ Yes ☐ No

4.05 **How often do you suffer from sleeplessness?** (tick once)

☐ Never, or just a few times a year
☐ 1-3 times a month
☐ Approximately once a week
☐ More than once a week

If you suffer from sleeplessness monthly or more often, what time of the year does it affect you most? (Put one or more ticks)

☐ No particular time
☐ Polar night time
☐ Midnight sun time
☐ Spring and autumn

4.06 **Have you had difficulty sleeping during the past couple of weeks?**

☐ Not at all
☐ No more than usual
☐ Rather more than usual
☐ Much more than usual

4.07 **Have you during the last two weeks felt unhappy and depressed?**

☐ Not at all
☐ No more than usual
☐ Rather more than usual
☐ Much more than usual

4.08 **Have you during the last two weeks felt unable to cope with your difficulties?**

☐ Not at all
☐ No more than usual
☐ Rather more than usual
☐ Much more than usual

4.09 **Below, please answer a few questions about your memory:** (tick once for each question)

	Yes	No
Do you think that your memory has declined?	☐	☐
Do you often forget where you have placed your things?	☐	☐
Do you have difficulties finding common words in a conversation?	☐	☐
Have you problems performing daily tasks you used to master?	☐	☐
Have you been examined for memory problems?	☐	☐

If YES to at least one of the first four questions above: Is this a problem in your daily life?

☐ Yes ☐ No

4.10 **Have you during the last last year suffered from pain and/or stiffness in muscles or joints in your neck/shoulders lasting for at least 3 consecutive months?**
(tick once for each line)

	No complaint	Little complaint	Severe complaint
Neck, shoulders	☐	☐	☐
Arms, hands	☐	☐	☐
Upper part of the back	☐	☐	☐
The lumbar region	☐	☐	☐
Hips, leg, feet	☐	☐	☐
Other places	☐	☐	☐

4.11 **Have you suffered from pain and/or stiffness in muscles or joints during the last 4 weeks?** (tick once for each line)

	No complaint	Little complaint	Severe complaint
Neck, shoulders	☐	☐	☐
Arms, hands	☐	☐	☐
Upper part of the back	☐	☐	☐
The lumbar region	☐	☐	☐
Hips, leg, feet	☐	☐	☐
Other places	☐	☐	☐

4.12 **Have you ever had:**

	Yes	No	Age last time
Fracture in the wrist/forearm?	☐	☐	☐☐
Hip fracture?	☐	☐	☐☐

4.13 **Have you been diagnosed with arthrosis by a physician?**
☐ Yes ☐ No

4.14 **Do you have or have you ever had some of the following:**

	Never	Some	Much
Nickel allergy	☐	☐	☐
Pollen allergy	☐	☐	☐
Other allergies	☐	☐	☐

4.15 **Have you ever experienced infertility for more than 1 year?**
☐ Yes ☐ No

If Yes: was it due to:

	Yes	No	Do not know
A condition concerning you?	☐	☐	☐
A condition concerning your partner?	☐	☐	☐

4.16 **To which degree have you had the following complaints during the last 12 months?**

	Never	Some	Much
Nausea	☐	☐	☐
Heartburn/regurgitation	☐	☐	☐
Diarrhoea	☐	☐	☐
Constipation	☐	☐	☐
Alternating diarrhoea and constipation	☐	☐	☐
Bloated stomach	☐	☐	☐
Abdominal pain	☐	☐	☐

4.17 **If you have had abdominal pain or discomfort during the last year:**

	Yes	No
Was it located in your upper stomach?	☐	☐
Were you bothered as often as once a week or more during the last 3 months?	☐	☐
Do you feel symptoms relief after bowel movement?	☐	☐
Are the symptoms related to more frequent or rare bowel movements than normally?	☐	☐
Are the symptoms related to more loose or hard stool than normally?	☐	☐
Do the symptoms appear after a meal?	☐	☐

4.18 **Have you ever had:**

	Yes	No	Age last time
Gastric ulcer	☐	☐	☐☐
Duodenal ulcer	☐	☐	☐☐
Ulcer surgery	☐	☐	☐☐

4.19 **For women: Have you ever had a miscarriage?**
☐ Yes ☐ No ☐ Do not know
If Yes: number of times ☐☐

4.20 **For men: Have your partner ever had a miscarriage?**
☐ Yes ☐ No ☐ Do not know
If Yes: number of times ☐☐

4.21 **Is your diet gluten-free?**
☐ Yes ☐ No ☐ Do not know

4.22 **Have you been diagnosed with Dermatitis Herpetiformis (DH)?**
☐ Yes ☐ No ☐ Do not know

4.23 **Have you been diagnosed with coeliac disease, based on a biopsy from your intestine taken in a gastroscopy examination?**

☐ Yes ☐ No ☐ Do not know

4.24 **Do you have your natural teeth?**

☐ Yes ☐ No

4.25 **How many amalgam tooth fillings do you have/have you had?**

☐ 0 ☐ 1-5 ☐ 6-10 ☐ 10+

4.26 **Have you been suffering from headache <u>the last year</u>?**

☐ Yes ☐ No

If No: go to section 5, food habits

4.27 **What kind of headache are you suffering from?**

☐ Migraine ☐ Other headache

4.28 **How many days <u>per month</u> do you suffer from headache?**

☐ Less than one day
☐ 1-6 days
☐ 7-14 days
☐ More than 14 days

4.29 **Is the headache attacks <u>usually</u>:** (tick once for each line)

	Yes	No
Pounding/pulsatory pain	☐	☐
Pressing/tightening pain	☐	☐
Unilateral pain *(right or left)*	☐	☐

4.30 **What is the normal intensity of your headache attacks?**

☐ Mild *(do not hinder normal activity)*
☐ Moderate *(decrease normal activity)*
☐ Strong *(block normal activity)*

4.31 **What is the normal duration of the headache attacks?**

☐ Less than 4 hours
☐ 4 hours - 1 day
☐ 1-3 days
☐ More than 3 days

4.32 **If you suffer from headache, when during the year does it affect you most?** (tick one or more)

☐ No particular time
☐ Polar night time
☐ Midnight sun time
☐ Spring and/or Autumn

4.33 **Before or during the headache, do you have a temporary:**

	Yes	No
Visual disturbances? *(flickering, blurred vision, flashes of light)*	☐	☐
Unilateral numbness in your face or hand?	☐	☐
Aggravated pain by moderate physical activity?	☐	☐
Nausea and/or vomiting?	☐	☐

4.34 **Describe how many days you have been away from work or school during the <u>last month</u> due to headache?**

Number of days ☐☐

5. FOOD HABITS

5.01 **How often do you usually eat the following?** (tick once for each line)

	0-1 times per month	2-3 times per month	1-3 times per week	More than 3 times per week
Fresh water fish *(not farmed)*	☐	☐	☐	☐
Salt water fish *(not farmed)*	☐	☐	☐	☐
Farmed fish *(salmon, trout, char)*	☐	☐	☐	☐
Tuna fish *(fresh or canned)*	☐	☐	☐	☐
Fish bread spread	☐	☐	☐	☐
Mussels, shells	☐	☐	☐	☐
The brown content in crabs	☐	☐	☐	☐
Whale or seal meat	☐	☐	☐	☐
Pluck (liver/kidney/heart) from reindeer or elk/moose	☐	☐	☐	☐
Pluck (liver/kidney/heart) from ptarmigan/grouse	☐	☐	☐	☐

5.02 **How many times during the year do/did you usually eat the following?** (number of times)

	In adulthood	In childhood
Mølje (cod or pollack meat, liver, and roe) *(Number of times per year)*	☐☐	☐☐
Sea gull's egg *(Number of eggs per year)*	☐☐	☐☐
Reindeer meat *(Number of times per year)*	☐☐	☐☐
Local mushroom and wild berries *(blueberries/lingonberries/cloudberries)* *(Number of times per year)*	☐☐☐	☐☐☐

5.03 **How many times per month do you eat canned (tinned) foods (from metal boxes)?**

Number ☐☐

5.04 **Do you take vitamins and/or mineral supplements?**

☐ Yes, daily ☐ Sometimes ☐ Never

5.05 **How often do you eat?**

	Never	1-3 times per month	1-3 times per week	4-6 times per week	1-2 times per day	3 times per day or more
Dark chocolate	☐	☐	☐	☐	☐	☐
Light chocolate/milk chocolate	☐	☐	☐	☐	☐	☐
Chocolate cake	☐	☐	☐	☐	☐	☐
Other sweets	☐	☐	☐	☐	☐	☐

5.06 **If you eat chocolate, how much do you usually eat each time?**
Compared with the size of a Kvikk-Lunsj sjokolade *(a chocolate brand in the market)* and describe how much do you eat in relation to it.

¼	½	1	1 ½	2	More than 2
☐	☐	☐	☐	☐	☐

5.07 **How often do you drink cocoa/hot chocolate?**

Never	1-3 times per month	1-3 times per week	4-6 times per week	1-2 times per day	3 times per day or more
☐	☐	☐	☐	☐	☐

6. ALCOHOL

6.01 **How often have you in the last year:**

	Never	Less than monthly	Monthly	Weekly	Daily or almost daily
Not been able to stop drinking alcohol when you have started?	☐	☐	☐	☐	☐
Failed to do what was normally expected of you because of drinking?	☐	☐	☐	☐	☐
Needed a drink in the morning to get yourself going after a heavy drinking session?	☐	☐	☐	☐	☐
Had feeling of guilt or remorse after drinking?	☐	☐	☐	☐	☐
Not been unable to remember what happened the night before because of your drinking?	☐	☐	☐	☐	☐

	Never	Yes, but not in the last year	Yes, during the last year
6.02 **Have you or someone else been injured because of your drinking?**	☐	☐	☐
**Has a relative, friend, physician, or other health care workers been concerned about your drinking or suggested you to cut down?**	☐	☐	☐

7. WEIGHT

7.01 **Have you involuntary lost weight during the last 6 months?**

☐ Yes ☐ No

If Yes: how many kilograms? .... [|]

7.02 **Estimate your body weight when you were 25 years old:**

Number of kilograms .... [| |]

7.03 **Are you satisfied with your present body weight?**

☐ Yes ☐ No

7.04 **What weight would you be satisfied with (your "ideal" weight)?**

Number of kilograms .... [| |]

8. SOLVENTS

8.01 **How many hours per week, do you do the following leisure- or professional activities:**

Automobile repair/paint, ceramic work, painting/varnishing/solvents, hair dressing, glazier, electrician. (Put 0 if you do not engage in such leisure or professional activities)

Number of hours per week on average [|]

8.02 **Do you use hair color preparations**

☐ Yes ☐ No

If Yes: How many times per year? .. [|]

9.01 **Have you ever experienced that diseases have been insufficiently examined or treated, and this had a serious consequence?**

- ☐ Yes, this has happened to me
- ☐ Yes, this has happened to a close relative *(child, parents, spouse)*
- ☐ No

If Yes, was it caused by?
(tick once or more):

- ☐ general practitioner
- ☐ emergency medical doctor
- ☐ private practising specialist
- ☐ hospital doctor
- ☐ other health personnel
- ☐ alternative practitioner
- ☐ more than one person due to deficient routines and interaction

9.02 **Have you ever felt persuaded to accept an examination or treatment that you did not want?**

☐ Yes ☐ No

If Yes, do you think this has had unfortunate consequences for your health?

☐ Yes ☐ No

9.03 **Have you ever complained about a treatment you have received?**

- ☐ Have never had a reason for complaining
- ☐ Have considered complaining, but did not do
- ☐ Have complained verbally
- ☐ Have complained in writing

9.04 **How long have you had your current general practitioner/other physician?**

- ☐ Less than 6 months
- ☐ 6 to 12 months
- ☐ 12 to 24 months
- ☐ More than 2 years

9.05 **At the last visit to your GP, did you have a hard time to understand what the doctor(s) told you?** Answer on a scale from 0 to 10, where 0 = they were difficult to understand and 10 = they were always easy to understand

0	1	2	3	4	5	6	7	8	9	10
☐	☐	☐	☐	☐	☐	☐	☐	☐	☐	☐

9.06 **How would you rate the treatment or counselling, you got at your last visit to your GP?** Answer on a scale from 0 to 10, where 0 = worst treatment or counselling, and 10 = best treatment or counselling

0	1	2	3	4	5	6	7	8	9	10
☐	☐	☐	☐	☐	☐	☐	☐	☐	☐	☐

9.07 **During the last 12 months, how much of a problem, if any, was it to get a referral to special examinations (as x-ray, etc.) or to a specialist health care (private practising specialist or at hospital)?**

- ☐ Not relevant
- ☐ No problem
- ☐ Some problem
- ☐ Major problem

9.08 **During the last 12 months, how much of a problem, if any, was it to get a referral to physiotherapist, chiropractor, etc.?**

- ☐ Not relevant
- ☐ No problem
- ☐ Some problem
- ☐ Major problem

9.09 **Altogether, how much of a problem, if any, was it to get a referral to specialist health care?**

- ☐ Not relevant
- ☐ Very difficult
- ☐ Some difficulties
- ☐ Easy
- ☐ Very easy

9.10 **During the last 12 months, have you been examined or treated by the specialist health care?**

☐ Yes ☐ No

If Yes, did you have a difficult time to understand what the doctor(s) told you? Answer on a scale from 0 to 10, where 0 = they were difficult to understand and 10 = they were always easy to understand

0	1	2	3	4	5	6	7	8	9	10
☐	☐	☐	☐	☐	☐	☐	☐	☐	☐	☐

9.11 **How would you rate the treatment or counselling you got at your last visit to a specialist?** Answer on a scale from 0 to 10, where 0 = worst treatment or counselling, and 10 = best treatment or counselling

0	1	2	3	4	5	6	7	8	9	10
☐	☐	☐	☐	☐	☐	☐	☐	☐	☐	☐

1.

9.12 **Have you ever, <u>previous to the year 2002</u>, had an operation at a hospital or a specialist clinic?**

☐ Yes ☐ No

9.13 **Have you, during the <u>last 12 months</u>, used herbal or natural medicine?**

☐ Yes ☐ No

9.14 **Have you, during the <u>last 12 months</u>, used meditation, yoga, qi gong or thai chi as self-treatment?**

☐ Yes ☐ No

10. USE OF ANTIBIOTICS

10.01 **Have you used antibiotics during <u>the last 12 months</u>?** (all penicillin-like medicine in the form of tablets, syrups or injections)

☐ Yes ☐ No ☐ Do not remember

If YES: What did you get the treatment for?
Have you taken many antibiotic treatments, tick for each treatment.

	Treatment 1	Treatment 2	Treatment 3	Treatment 4	Treatment 5	Treatment 6
• Urinary tract infection *(bladder infection, cystitis)*	☐	☐	☐	☐	☐	☐
• Respiratory tract infection *(ear, sinus, throat or lung infection, bronchitis)*	☐	☐	☐	☐	☐	☐
• Other	☐	☐	☐	☐	☐	☐
Treatment duration: number of days	☐☐	☐☐	☐☐	☐☐	☐☐	☐☐

How did you acquire the antibiotics for treatment?
Have you acquired many treatments, tick for each one.

With prescription from a physician/dentist	☐	☐	☐	☐	☐	☐
Without contacting a physician/without prescription:						
· Purchase from a pharmacy abroad	☐	☐	☐	☐	☐	☐
· Purchase over the internet	☐	☐	☐	☐	☐	☐
· Remnants from earlier treatment at home	☐	☐	☐	☐	☐	☐
· From family/friends	☐	☐	☐	☐	☐	☐
· Other ways	☐	☐	☐	☐	☐	☐

10.02 **Do you presently have antibiotics at home?**

☐ Yes ☐ No

If YES:is this after an agreement with your physician for treatment of chronic or frequently recurring disease?

☐ Yes ☐ No

If No: how did you acquire this antibiotic? (Multiple ticks are possible)

- Purchased from a pharmacy abroad ☐
- Purchased over the internet ☐
- Remnants from earlier treatment ☐
- From family/friends ☐
- Other ways ☐

10.03 **Would you consider using antibiotics without consulting your physician?**

☐ Yes ☐ No

If YES: which conditions would you treat in such situation? (multiple ticks are possible)

- Common cold ☐
- Cough ☐
- Bronchitis ☐
- Sore throat ☐
- Sinusitis ☐
- Fever ☐
- Influenza ☐
- Ear infection ☐
- Diarrhoea ☐
- Urinary tract infection ☐
- Other infections ☐

11. YOUR CIRCADIAN RHYTHM

We will ask you some questions about your sleeping habits

11.01 **Have you worked in a shift work schedule during the last 3 months?**

☐ Yes ☐ No

11.02 **Number of days per week which you cannot freely choose when you sleep (e.g. work days)?**

0 ☐ 1 ☐ 2 ☐ 3 ☐ 4 ☐ 5 ☐ 6 ☐ 7 ☐

Then I go to bed at .... __ __ : __ __

I get ready to fall asleep at .... __ __ : __ __

Number of minutes I need to fall asleep .... __ __

I wake up at .... __ __ : __ __

With help of: ☐ Alarm clock ☐ External stimulus *(noise, family members etc.)* ☐ By myself

Number of minutes I need to get up .... __ __

11.03 **Number of days per week which you can freely choose when you sleep** (e.g. free days or holidays)

0 ☐ 1 ☐ 2 ☐ 3 ☐ 4 ☐ 5 ☐ 6 ☐ 7 ☐

Then I go to bed at .... __ __ : __ __

I get ready to fall asleep at .... __ __ : __ __

Number of minutes I need to fall asleep .... __ __

I wake up at .... __ __ : __ __

With help of: ☐ Alarm clock ☐ External stimulus *(noise, family members etc.)* ☐ By myself

Number of minutes I need to get up .... __ __

12. SKIN AND DERMATOLOGY

12.01 **How often do you usually take a shower or a bath?** (tick once)

- ☐ 2 or more times daily
- ☐ 1 time daily
- ☐ 4-6 times per week
- ☐ 2-3 times per week
- ☐ Once a week
- ☐ Less than once a week

12.02 **How often do you usually wash your hands with soap <u>daily</u>?** (tick once)

- ☐ 0 times
- ☐ 1-5 times
- ☐ 6-10 times
- ☐ 11-20 times
- ☐ More than 20 times

12.03 **Have you ever taken any antibiotics (penicillin and penicillin-like medicines) because of a skin disease, for example infected eczema, acne, non-healing leg ulcers, recurrent abscess?**

☐ Yes ☐ No

If Yes: How many times in average per year did you take antibiotics during the period you were most affected (tick once)

☐ 1-2 ☐ 3-4 ☐ More than 4 times

12.04 **Have you or have you ever had the following skin disorders?** (tick once for each line)

	Yes	No
Psoriasis	☐	☐
Atopic eczema (children's eczema)	☐	☐
Recurrent hand eczema	☐	☐
Recurrent pimples/spots for several months	☐	☐
Leg or foot ulcer that did not heal for 3-4 weeks	☐	☐

If YES on the question concerning leg and/or foot ulcer, do you have any leg ulcer today?

☐ Yes ☐ No

12.05 **Have you often or always any of the following complaints?** (tick once for each line)

	Yes	No
Swelling in the ankles or legs, particularly in the evenings	☐	☐
Varicose veins	☐	☐
Eczema (red, itchy rash) on your legs	☐	☐
Leg pain that is getting worse when you are walking and is relieved when you are standing still	☐	☐

12.06 **Have you ever had the following diagnoses by a <u>physician</u>?** (tick once for each line)

	Yes	No
Psoriasis	☐	☐
Atopic eczema	☐	☐
Rosacea	☐	☐

12.07 **Have you recurring large acne/abscesses that are tender/painful and often form scars in the following places?** (tick once for each line)

	Yes	No
Armpits	☐	☐
Under the breasts	☐	☐
Stomach groove/the navel	☐	☐
Around the genitalia	☐	☐
Around the anus	☐	☐
The groin	☐	☐

If Yes: Have you ever visited a physician because of abscesses?

☐ Yes ☐ No

If Yes, did you get any of the following treatments? (tick once for each line)

	Yes	No
Antibiotic ointment	☐	☐
Antibiotic tablets	☐	☐
Surgical drainage	☐	☐
A larger surgical intervention including skin removal	☐	☐
Surgical laser treatment	☐	☐

Follow-up questions

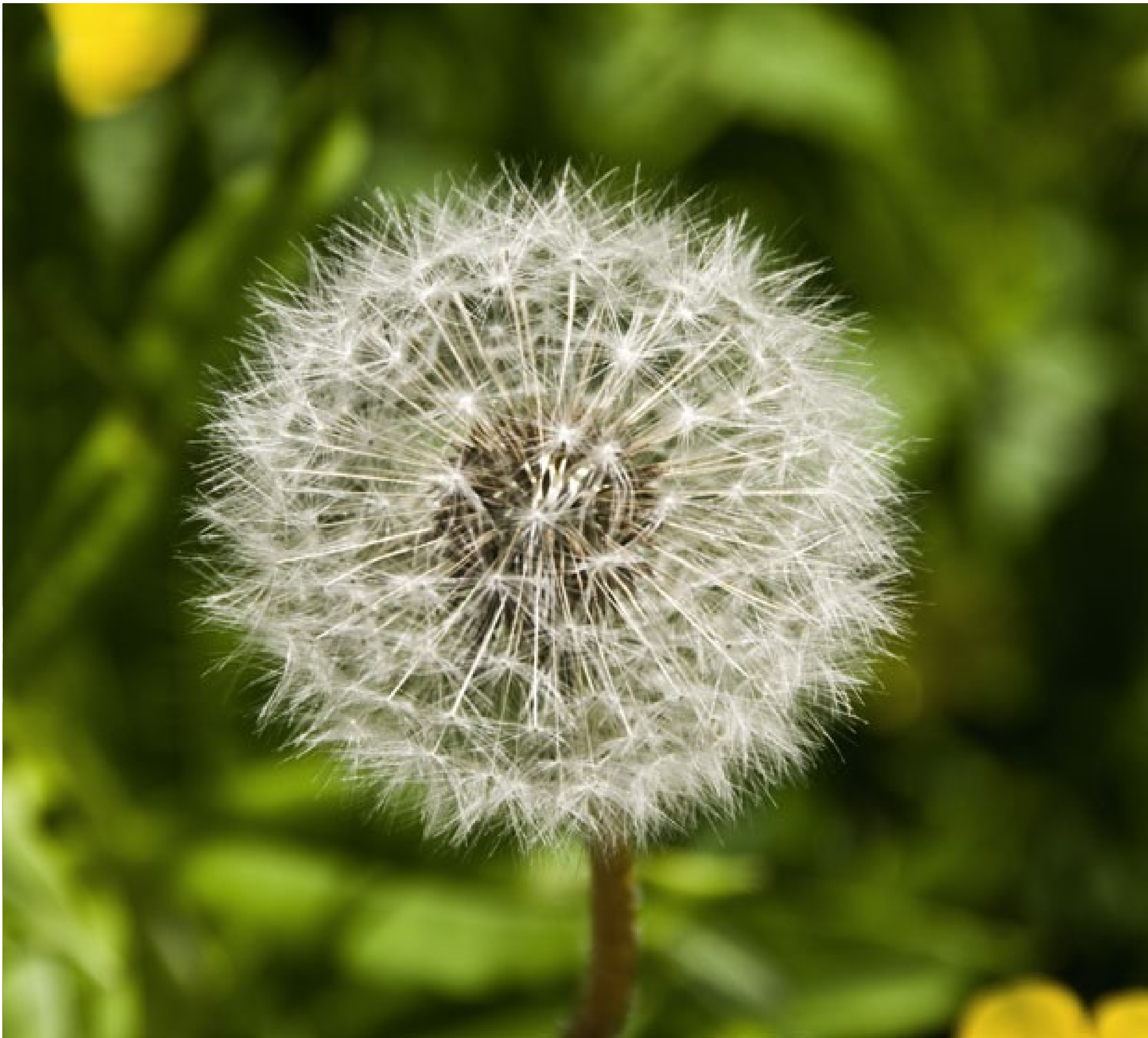

INFORMATION TO FOLLOW-UP QUESTIONS

The following pages with questions should not be answered by everybody. If you have answered yes to one or more of questions below, we ask you to move on to the follow-up questions on the topic or topics you have answered yes to. The first four topics are from the first questionnaire and the last question is from this form.

We have for the sake of simplicity highlighted topics with different colours so that you will find the questions that applies to you.

If you answered YES to that you have: long-term or recurrent pain that has lasted for 3 months or more, please answer the questions on page 19 and 20. The margin is marked with green.

If you answered YES to that you have undergone any surgery during the last 3 years, please answer the questions on page 21 and 22. The margin is marked with purple.

If you answered YES to that you're working outdoors at least 25% of the time, or in facilities with low temperature, such as warehouse/industrial halls, please answer the questions on page 23. The margin is marked with red.

If you answered YES to that you have used non-prescription pain relievers, please answer questions on page 24. The margin is marked with orange.

If you answered YES to that you have or have ever had skin problems (such as psoriasis, atopic eczema, non-healing leg or foot ulcers, recurrent hand eczema, acne or abscesses), please answer the questions on page 25. The margin is marked with yellow.

If you have answered **NO** to these five questions, you are finished with your answers. The questionnaire is to be returned in the reply envelope you were given at the survey site. The postage is already paid.

Should you wish to give us written feedback on either the questionnaire or The Tromsø Study in general, you are welcome to that on page 26.

Do you have any questions, please contact us by phone or by e-mail. You can find the contact information on the back of the form. **THANK YOU** for taking the time to the survey and to answer our questions.

13. FOLLOW-UP QUESTIONS ON PAIN

You answered in the first questionnaire that you have protracted or constantly recurrent pain that has lasted for 3 months or more. Here, we ask you to describe the pain a little closer.

13.01 **How long have you had this pain?**

Number of years ☐ months ☐

13.02 **How often do you have this pain?**

- ☐ Every day
- ☐ Once a week or more
- ☐ Once a month or more
- ☐ Less than once a month

13.03 **Where does it hurt?** (Tick for all locations where you have protracted or constantly recurrent pain)

- ☐ Head/face
- ☐ Jaw/temporo-mandibular joint
- ☐ Neck
- ☐ Back
- ☐ Shoulder
- ☐ Arm/elbow
- ☐ Hand
- ☐ Hip
- ☐ Thigh/knee/leg
- ☐ Ankle/foot
- ☐ Chest/breast
- ☐ Stomach
- ☐ Genitalia /reproductive organs
- ☐ Skin
- ☐ Other location

13.04 **What do you believe is the cause of the pain?** (Tick for all known causes)

- ☐ Accident /acute injury
- ☐ Long-term stress
- ☐ Surgical intervention/operation
- ☐ Herniated disk *(prolapse)* /lumbago
- ☐ Whiplash
- ☐ Migraine/headache
- ☐ Osteoarthritis
- ☐ Rheumatoid arthritis
- ☐ Bechterews syndrome
- ☐ Fibromyalgia
- ☐ Angina pectoris
- ☐ Poor blood circulation
- ☐ Cancer
- ☐ Nerve damage/neuropathy
- ☐ Infection
- ☐ Herpes zoster
- ☐ Another cause *(describe below)*
- ☐ Don't know

Describe the other cause:

....

13.05 **Which kind of treatment have you received for the pain?** (Tick for all types of pain treatments you have received)

- ☐ No treatment
- ☐ Analgesic medications/painkillers
- ☐ Physiotherapy/chiropractic treatment
- ☐ Treatment at a pain clinic
- ☐ Surgery
- ☐ Psycho-educative/relaxation training/ psychotherapy
- ☐ Acupuncture
- ☐ Complimentary and alternative medicine *(homeopathy, healing, aromatherapy, etc.*
- ☐ Other treatment

13.06 **On a scale of 0 to 10, where 0 corresponds to no pain and 10 corresponds to the worst possible pain you can imagine:**

		0	1	2	3	4	5	6	7	8	9	10	
How strong would you say that the pain usually is?	No pain	☐	☐	☐	☐	☐	☐	☐	☐	☐	☐	☐	Worst imaginable pain
How strong is the pain when it is in its strongest Intense?	No pain	☐	☐	☐	☐	☐	☐	☐	☐	☐	☐	☐	Worst imaginable pain
To what degree does the pain interfere with your sleep?	No effect	☐	☐	☐	☐	☐	☐	☐	☐	☐	☐	☐	Impossible to sleep
To what degree does the pain interfere with performing common activities at home and at work?	No effect	☐	☐	☐	☐	☐	☐	☐	☐	☐	☐	☐	Can not do anything

14. FOLLOW-UP QUESTIONS ON SURGERY

In the first questionnaire you answered that you have undergone an operation during the last 3 years.

14.01 **How many times have you undergone surgery during the last 3 years?**

Number ☐

Below, please describe the operation. If you have undergone several operations during the last 3 years, these questions concern the last surgery you underwent.

14.02 **Where in your body did you have surgery?**
(If you were operated simultaneously in several places in the body, tick more than once)

Surgery in the head/neck/back
- Head/face ☐
- Neck/throat ☐
- Back ☐

Surgery in the chest
- Heart ☐
- Lungs ☐
- Breasts ☐
- Another surgery in the chest region ☐

Surgery in the stomach/pelvis
- Stomach/intestines ☐
- Inguinal hernia ☐
- Urinary tract/reproductive organs ☐
- Gall bladder/biliary tract ☐
- Another surgery in the stomach/pelvis ☐

Surgery in the hip/legs
- Hip/thigh ☐
- Knee/leg ☐
- Ankle/foot ☐
- Amputation ☐

Surgery in the shoulder and arm
- Shoulder/overarm ☐
- Elbow/underarm ☐
- Hand ☐
- Amputation ☐

14.03 **Reason for the surgery:**
- Acute illness/trauma ☐
- Planned non-cosmetic operation ☐
- Planned cosmetic operation ☐

14.04 **Where did you have the surgery?**
- The hospital in Tromsø ☐
- The hospital in Harstad ☐
- Other public hospital ☐
- Private clinic ☐

14.05 **How long time is it since you had surgery?**

Number of years ☐ Months ☐

14.06 **Do you have reduced sensitivity in an area near the surgical scar?**

☐ Yes ☐ No

14.07 **Are you hypersensitive to touch, heat or cold in an area near the surgical scar?**

☐ Yes ☐ No

14.08 **Does slight touch from clothes, showering or similar cause discomfort/pain?**

☐ Yes ☐ No

14.09 **If you had pain at the site of surgery before you had surgery, do you have the same type of pain now?**

☐ Yes ☐ No

14.10 **The pain at the site of surgery:** Answer on a scale from 0 to 10, where 0=no pain and 10=worst pain you can imagine

	No pain	0	1	2	3	4	5	6	7	8	9	10	Worst imaginable pain
How strong pain did you have at the site of surgery <u>before</u> you had surgery		☐	☐	☐	☐	☐	☐	☐	☐	☐	☐	☐	
How strong pain do you normally have at the site of surgery now		☐	☐	☐	☐	☐	☐	☐	☐	☐	☐	☐	
How strong pain do you normally have at the site of surgery when it is most intense		☐	☐	☐	☐	☐	☐	☐	☐	☐	☐	☐	

15. FOLLOW-UP QUESTIONS ABOUT WORK IN COLD ENVIRONMENT

In the first questionnaire you answered yes to that you work in cold environments. Here are some follow-up questions that we hope you will answer.

15.01 **Do you feel cold at work?**

- ☐ Yes, often
- ☐ Yes, sometimes
- ☐ No, never

15.02 **For how long have you been exposed to cold air below 0°C during the last winter?**

Leisure/hobbies *(hours/week)*	☐
Work *(hours/week)*	☐
Outdoors, with suitable clothing *(hours/week)*	☐
Outdoors, without suitable clothing *(hours/week)*	☐
Indoors, with no heating *(hours/week)*	☐
In cold, with wet clothing *(hours/week)*	☐
Contact with cold objects/tools *(hours/week)*	☐

15.03 **What ambient temperature prevents you from:**

	Under °C
Working outdoors	☐
Training outdoors	☐
Performing other activities outdoors	☐

15.04 **Have you during the last 12 months had a frostbite with blisters, sores or skin injury?**

☐ Yes ☐ No

If Yes, how many times? ☐

15.05 **Have you had itching and/or rash in relation to cold exposure?**

☐ Yes ☐ No

15.06 **Have you during the last 12 months had an accident where cold has been involved, and which required medical treatment?**

	Yes	No
At work	☐	☐
In leisure time	☐	☐

15.07 **Do you experience any of the following symptoms while you are in a cold environment? If so, at what temperature do the symptoms occur?**

	Yes	No	Under °C
Breathing problems	☐	☐	☐
Wheezy breathing	☐	☐	☐
Mucus secretion from lungs	☐	☐	☐
Chest pain	☐	☐	☐
Disturbance in heart rhythm	☐	☐	☐
Impaired blood circulation in hands/feet	☐	☐	☐
Visual disturbance *(short term/transient)*	☐	☐	☐
Migraine *(short term/transient)*	☐	☐	☐
Fingers turning white *(short term/transient)*	☐	☐	☐
Fingers turning blue-red *(short term/transient)*	☐	☐	☐

15.08 **How does cold environments and cold-related symptoms influence your performance?**

	Decrease	No effect	Improve
Concentration	☐	☐	☐
Memory	☐	☐	☐
Finger sensitivity *(feeling)*	☐	☐	☐
Finger dexterity *(motor)*	☐	☐	☐
Control of movement *(for example tremor)*	☐	☐	☐
Heavy physical work	☐	☐	☐
Long-lasting physical work	☐	☐	☐

16. USE OF NON-PRESCRIPTION PAINKILLERS

In the first questionnaire you answered that you had used non-prescription painkillers (analgesics) in the last 4 weeks. Here are some follow-up questions we hope you will answer.

16.01 **What types of non-prescription painkillers have you used?**

Paracetamol: *(Pamol, Panodil, Paracet, Paracetamol, Pinex)*

- ☐ Not used
- ☐ Less than every week
- ☐ Every week, but not daily
- ☐ daily

How much do you usually take daily when you use these medicines? *(number of tablets, suppositories)* .... ☐☐

Acetylsalicylates: *(Aspirin,Dispril, Globoid)*

- ☐ Not used
- ☐ Less than every week
- ☐ Every week, but not daily
- ☐ Daily

How much do you usually take daily when you use these medicines? *(number of tablets)* .... ☐☐

Ibuprofen: *(Ibumetin, Ibuprofen, Ibuprox, Ibux)*

- ☐ Not used
- ☐ Less than every week
- ☐ Every week, but not daily
- ☐ Daily

How much do you usually take daily when you use these medicines? *(number of tablets, suppositories)* .... ☐☐

Naproxen: *(Ledox, Naproxen)*

- ☐ Not used
- ☐ Less than every week
- ☐ Every week, but not daily
- ☐ Daily

How much do you usually take daily when you use these medicines? *(number of tablets)* .... ☐☐

Phenazone with caffeine: *(Antineuralgica, Fanalgin, Fenazon-koffein, Fenazon-koffein sterke)*

- ☐ Not used
- ☐ Less than every week
- ☐ Every week, but not daily
- ☐ daily

How much do you usually take daily when you use these medicines? *(number of tablets)* .... ☐☐

16.02 **For which complaints do you use non-prescription painkillers?** (multiple ticks are possible)

- ☐ Headache
- ☐ Menstrual discomfort
- ☐ Migraine
- ☐ Back pain
- ☐ Muscle/joint pain
- ☐ Tooth pain
- ☐ Other

16.03 **Do you think you have experienced side effects of some of the medicines?** (tick once for each line)

	Yes	No
Paracetamol	☐	☐
Acetylsalicylates	☐	☐
Ibuprofen	☐	☐
Naproxen	☐	☐
Phenazone with caffeine	☐	☐

16.04 **Where do you usually purchase painkillers?**

- ☐ Pharmacy
- ☐ Grocery
- ☐ Petrol stations
- ☐ Abroad
- ☐ Internet

16.05 **Do you combine the treatment with the use of painkillers on prescription?**

☐ Yes ☐ No

17. FOLLOW-UP QUESTIONS ABOUT SKIN DISEASES

On page 15 in this questionnaire you answered that you have or have had a skin disease. Here are some follow-up questions we hope you will answer.

Answer on a scale from 0 to 10, where 0 corresponds to no symptoms and 10 correspond to worst imaginable complaints. If you answered YES to that you have or have had:

	No complaint 0	1	2	3	4	5	6	7	8	9	Worst imaginable complaints 10
17.01 **Psoriasis**											
· How much are you affected by your psoriasis today?	☐	☐	☐	☐	☐	☐	☐	☐	☐	☐	☐
· How much are you affected by your psoriasis when it is most severe?	☐	☐	☐	☐	☐	☐	☐	☐	☐	☐	☐
17.02 **Atopic eczema**											
· How much are you affected by your atopic eczema today?	☐	☐	☐	☐	☐	☐	☐	☐	☐	☐	☐
· How much are you affected by your atopic eczema when it is most severe?	☐	☐	☐	☐	☐	☐	☐	☐	☐	☐	☐
17.03 **Hand eczema**											
· How much are you affected by your hand eczema today?	☐	☐	☐	☐	☐	☐	☐	☐	☐	☐	☐
· How much are you affected by your hand eczema when it is most severe?	☐	☐	☐	☐	☐	☐	☐	☐	☐	☐	☐
17.04 **Acne**											
· How much are you affected by your acne today?	☐	☐	☐	☐	☐	☐	☐	☐	☐	☐	☐
· How much are you affected by your acne when it is most severe?	☐	☐	☐	☐	☐	☐	☐	☐	☐	☐	☐
17.05 **Abscesses**											
· How much are you affected by your abscesses today?	☐	☐	☐	☐	☐	☐	☐	☐	☐	☐	☐
· How much are you affected by your abscesses when it is most severe?	☐	☐	☐	☐	☐	☐	☐	☐	☐	☐	☐

17.06 **Here is a list of factors that might trigger or exacerbate abscesses, tick for what you think apply to you:**

	Yes	No
Stress/psychological strain	☐	☐
Narrow/tight clothing	☐	☐
Menstrual periods	☐	☐
Pregnancy	☐	☐
Other	☐	☐

17.07 **How many episodes of abscesses do you usually have per year?** (tick once)

☐ 0-1 ☐ 4-6
☐ 2-3 ☐ More than 6

17.08 **How old were you when you got abscesses for the first time?**

☐ 0-12 years ☐ 26-35 years
☐ 13-19 years ☐ 36-50 years
☐ 20-25 years ☐ Older than 50 years

17.09 **If you no longer have abscesses, how old were you when it disappeared?**

☐ 0-12 years ☐ 26-35 years
☐ 13-19 years ☐ 36-50 years
☐ 20-25 years ☐ Older than 50 years

FEEDBACK

Should you wish to give us a written feedback on either the questionnaire or The Tromsø Study in general, you are welcome to do it here:

Thank you for your help

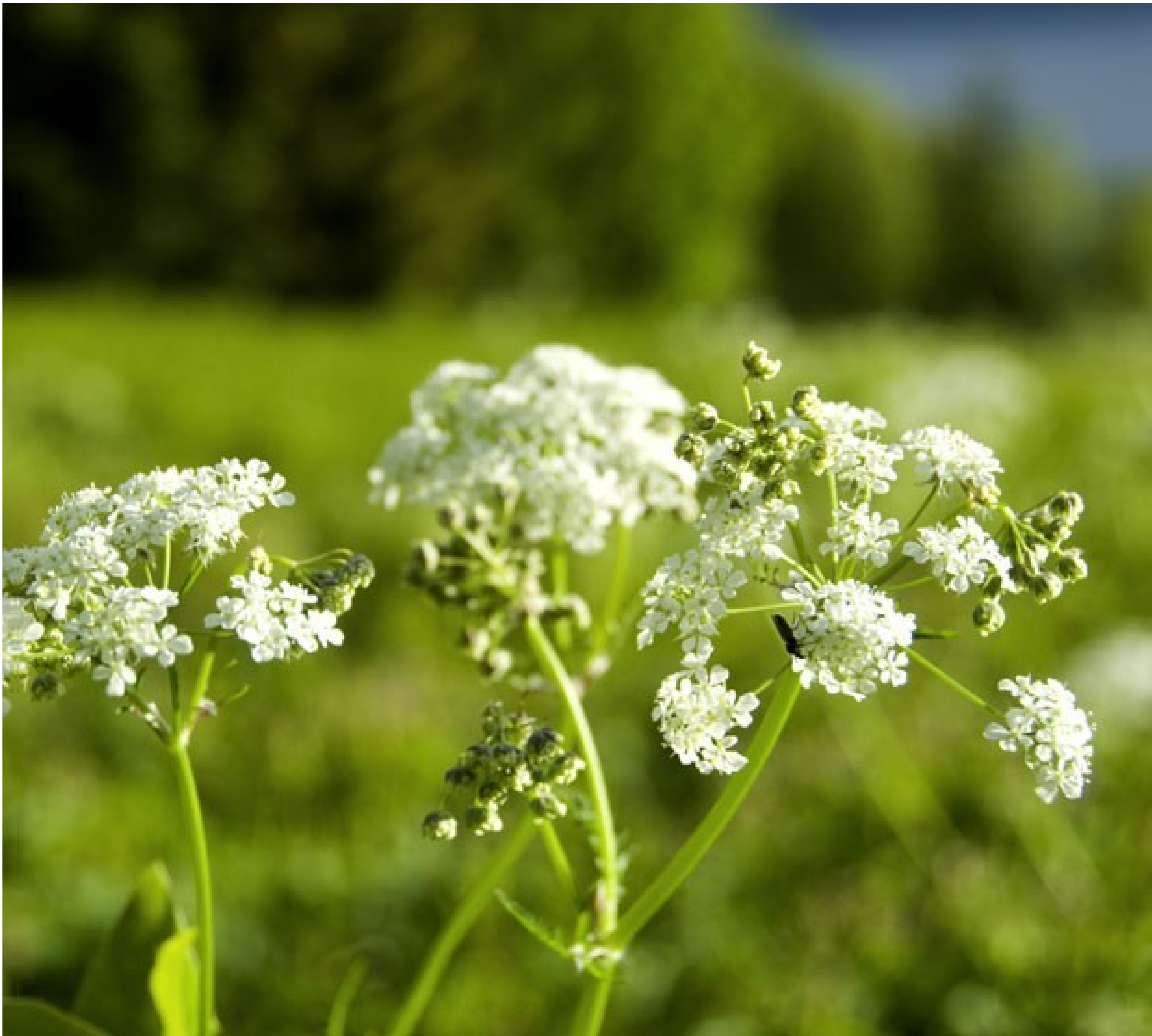

LUNDBLAD MEDIA AS - FOTO: TORGER GRYTÅ

Tromsø-undersøkelsen

The Tromsø Study
Department of community medicine, University of Tromsø
9037 TROMSØ
Telephone: 77 64 48 16
Telefax: 77 64 48 31
Email: tromsous@ism.uit.no
www.tromso6.no

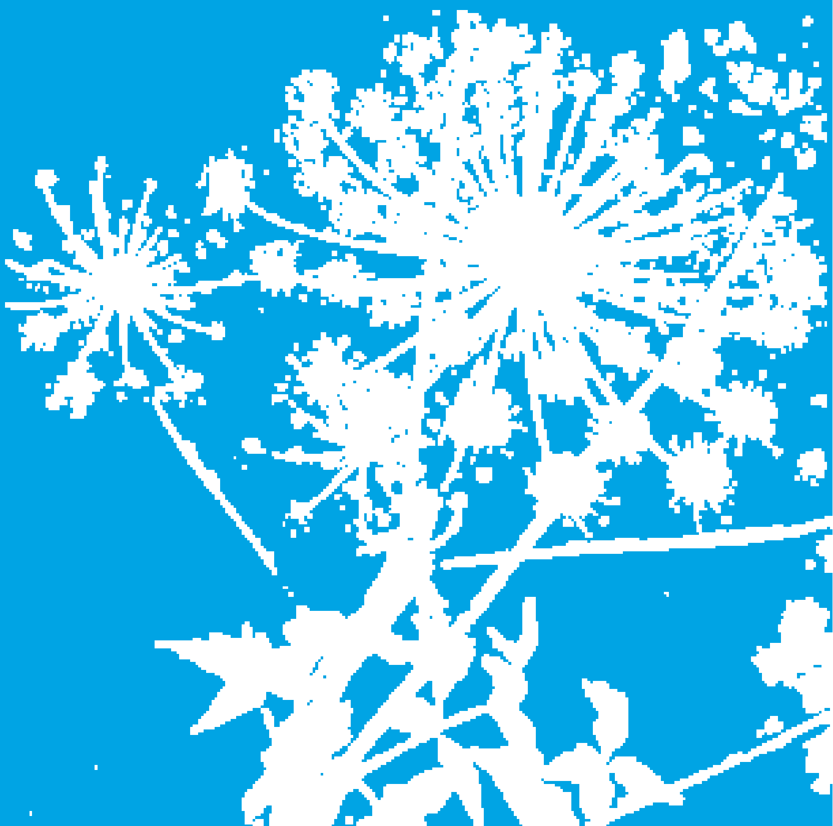

Supplement: Additional file 2 — Extended questionnaire. Delivered to persons attending the study. [file 1472-6963-13-46-S2.pdf]
